# Compartment Syndrome with Rhabdomyolysis in a Marathon Runner

**DOI:** 10.5811/cpcem.2018.4.37957

**Published:** 2018-06-12

**Authors:** Alaina Brinley, Bharath Chakravarthy, Douglas Kiester, Wirachin Hoonpongsimanont, C. Eric McCoy, Shahram Lotfipour

**Affiliations:** * University of California, Irvine, Department of Emergency Medicine, Orange, California; † University of California, Irvine, Department of Orthopedics, Orange, California

## Abstract

A 38-year-old female seasoned marathon runner presented to the emergency department (ED) with increasing right lower extremity pain after running two mid-distance races in one weekend. The patient had previously run many two-day races and longer distances, but recently had gained weight and had not been training. This case report details her presenting symptoms, evaluation, review of the literature, and treatment with attention to the factors that led to the development of her pathologies.

## INTRODUCTION

Acute compartment syndrome is a serious disease, which can result in severe morbidity and even mortality. Outcomes are progressively worse with delayed recognition of the diagnosis and the associated delayed treatment. The vast majority of compartment syndrome-associated injuries occur in relation to high-velocity trauma, fractures, and crush injuries. This article discusses the less-common cause: overuse and exercise. Many athletes are accustomed to significant muscle pain after training and events, which may delay the diagnosis. In addition, this patient used opiate painkillers after the races, which may have further delayed her presentation. To our knowledge, there is no previous report detailing a case of compartment syndrome after exercise, with concurrent use of opiate painkillers by the patient. This case report discusses a female recreational runner who presented to a tertiary care emergency department after two mid-distance running events in one weekend.

## CASE REPORT

A 38-year-old female presented with right foot, ankle, and calf pain. Her past history was remarkable for a 55-pound weight gain in the prior six months due to being sedentary, and she had a history of meralgia paresthetica of her right lower extremity after a motor vehicle collision. The patient stated that she had completed a 10K race (6.2 miles) two days prior to presentation and a half marathon (13.1 miles) one day prior to presentation when she noticed her right calf started “seizing up” during the second race. She then started to experience pain on the dorsal aspect of her right foot. The pain progressively worsened over the next 24 hours until she could no longer bear weight on the right lower extremity without severe pain. The pain was worse on the posterior/lateral leg and lateral ankle with associated foot numbness and burning in the sensory distribution of L2-S1. Her sensation was intact to light touch in the sensory distribution of L2-S1 and throughout her lower extremity, despite perceived numbness to the dorsal aspect of her foot and lateral calf. The pulses in her leg were 2+ in femoral, dorsalis pedis, and posterior tibial locations. She also cited intermittent pulling and tightness at rest and with active motion.

She had attempted her normal post-race remedies including ice, hot baths, ibuprofen and hydrocodone/paracetamol. Nothing improved her pain. Stepping on the leg, moving, or touching the leg was extremely painful. Physical exam showed normal vital signs and was significant for an uncomfortable appearing, overweight woman. She allowed a limited physical exam; however, she refused to move the extremity actively or passively.

A radiograph did not show a fracture and ultrasound did not show a deep venous thrombosis. Her creatinine kinase was 5533 (30 – 223 U/L). Intravenous fluid resuscitation was immediately initiated. Given that her pain seemed out of proportion to the exam, orthopedic surgery was consulted. Upon orthopedic evaluation, the patient was diagnosed with compartment syndrome based on the physical exam. She was taken to surgery for emergent lateral/anterior/superficial and deep posterior compartment (four-compartment) fasciotomy. Vacuum-assisted closure was placed on the fasciotomy wounds. A delayed primary closure of all of her wounds was done on postoperative day three. She was discharged the following day.

## DISCUSSION

Compartment syndrome is a relatively rare condition. The vast majority of cases occur in individuals who have had fractures, high-velocity trauma, or crush injuries.^1^ However, compartment syndrome has also been reported with exercise, overuse injuries and post operatively. Many of the postoperative cases are due to poor positioning on the operating table of an extremity that was not part of the surgery.

In this case, the patient was a trained marathoner who usually participated in multiple, long-distance running races per year. Her deconditioning coupled with significant weight gain were major factors rendering her more susceptible to muscle overuse injury, which subsequently led to the development of her compartment syndrome. Additionally, the patient’s narcotic use likely contributed to delayed presentation. There is no previous literature describing the use of narcotics by endurance athletes for post-workout pain. This represents a unique presentation of compartment syndrome and is significant given the current national focus on the opioid epidemic and overuse of narcotic pain prescriptions. The patient presented in this case was using narcotics inappropriately, which provides another example of a risk for over-prescription of narcotics by providers.

Each muscular compartment is a tightly-closed anatomic container that has very little capacity to expand. After an injury, the muscle begins to swell due to increased blood flow to the injured area. In some cases, the swelling is extreme and leads to microvascular and venous dysfunction and collapse. This in turn leads to decreased venous outflow and further engorgement of the muscle, which then causes a continuous rise in compartment pressure. This can also lead to extravascular fluid collections and edema. When the compartment pressure rises to a level higher than the blood pressure, the muscle becomes ischemic and then necrotic, resulting in rhabdomyolysis, which then results in myoglobinemia. It is the myoglobinemia that interferes with kidneys and cardiac function and is the major cause of death in these cases. This process was already well underway in our patient at the time of presentation, which is why her creatinine kinase was so high. Although compartment syndrome is a clinical diagnosis, a measured value of compartment pressure above 30 mm Hg above the diastolic blood pressure can also make the diagnosis. Modalities of treatment include conservative monitoring of compartment pressures and emergent fasciotomy.^2^,^3^

**CPC-EM Capsule**

What do we already know about this clinical entity?

Compartment syndrome is a well-characterized disease that usually occurs secondary to trauma, fracture, or overuse injury.

What makes this presentation of disease reportable?

This patient was a seasoned marathoner who had become deconditioned, ran two races in as many days, and then used narcotic pain medication to treat her post-run pain.

What is the major learning point?

Narcotic medication should not be used for post-exertional pain as it may mask injuries, such as compartment syndrome, and delay presentation.

How might this improve emergency medicine practice?

Emergency medicine practitioners should be cautious prescribing opiates as patients may use the medication for conditions other than the intended condition.

The treatment for most acute compartment syndrome is emergent decompression of each involved compartment. This is managed surgically by making a longitudinal incision into the fascia. In a successful compartment syndrome fasciotomy, the pressurized muscle will decompress with an immediate color change noted from a dark purple to a bright red. If the compartment is not successfully treated, the muscle develops scar tissue, the nerves die, and circulation is permanently compromised. Permanent disability can result including extremity motor/sensory deficits, loss of all motion, even passive motion, and severe dysesthesias. Any of these conditions can lead to the extremity being amputated.^4^

Glass et al. completed a systematic review of compartment syndrome, finding that delays in detection of compartment syndrome resulted in the most severe morbidity and mortality. The severe side effects of compartment syndrome (i.e., death and amputation) may be prevented by emergent fasciotomy performed by an orthopedic surgeon or qualified emergency physician; however, even after fasciotomy many patients still experience deficits such as foot drop, limb weakness, or sensory deficits. Some also progress to late amputations. Early diagnosis and treatment are the best predictors of good outcomes; however, there is no consensus in the literature regarding the best surgical method of treatment for compartment syndrome patients.^3^

Pain, or pain out of proportion to exam, is often the initial symptom indicating compartment syndrome pathology. Patients who are nonverbal as a result of their trauma, or pediatric patients, require close monitoring for physical signs of compartment syndrome as they may not be able to verbalize worsening pain.^1^ Other reports suggest compartment syndrome in lower extremities after harvesting bypass grafts and urologic procedures.^5^,^6^ These patients may also be under heavy sedation and post-operative analgesia, further complicating early diagnosis. However, young athletes are usually able to determine when their pain progresses to abnormal levels and will seek medical aid. However, in the case we have presented, this patient’s awareness of her condition may have been delayed by her use of narcotics for post-exercise pain.

Athletes may also develop chronic compartment syndrome where repetitive injury causes tissue and microvascular damage that leads to progressively elevated compartment pressures, rather than acute compartment pressures.^2^ Chronic exertional compartment syndrome may be treated conservatively with physical therapy or gait retraining; it may also be treated operatively, most often arthroscopically. Campano et al. report good outcomes in 84% of operatively-managed chronic exertional compartment syndrome patients.^7^,^8^

## CONCLUSION

A high index of suspicion is an important consideration in patients presenting with calf discomfort without trauma but having pain out of proportion to exam. Our patient had previous experience in athletic competitions; however, significant recent weight gain added risk to this otherwise-seasoned athlete. Additionally, her use of narcotics likely delayed her presentation. It would have been easy to attribute her pain after two relatively short races to muscle fatigue, sprain and stress from the physical exertion. The risk of missing compartment syndrome has very serious morbidity and medical consequences, particularly for athletes, which makes consideration of this entity a critical diagnosis for emergency physicians.

Documented patient informed consent and/or Institutional Review Board approval has been obtained and filed for publication of this case report.
